# Type 2 Diabetes Mellitus With Complex Necrotizing Otitis Externa, Skull Base Osteomyelitis, and Cranial Nerve Palsies: A Case Report

**DOI:** 10.7759/cureus.95710

**Published:** 2025-10-29

**Authors:** Manoj Kumar Mahadevaswamy Susheela, Ayoyimika O Okunlola, Syahmina Sufrian, Musla Mohamed, Cornelius Fernandez

**Affiliations:** 1 Diabetes and Endocrinology, United Lincolnshire Hospitals NHS Trust, Lincolnshire, GBR; 2 Internal Medicine, United Lincolnshire Hospitals NHS Trust, Lincolnshire, GBR; 3 Endocrinology, United Lincolnshire Hospitals NHS Trust, Lincolnshire, GBR

**Keywords:** cranial imaging, cranial nerve palsy, otitis externa, skull base osteomyelitis, type 2 diabetes mellitus

## Abstract

Necrotizing otitis externa (NOE) is a severe infection of the external auditory canal that can extend to the skull base. We report the case of a 67-year-old gentleman with insulin-dependent type 2 diabetes mellitus with suboptimal glycemic control who presented with a likely complex NOE. Initial findings included ciprofloxacin-resistant *Pseudomonas aeruginosa*, elevated inflammatory markers, and multiple cranial nerve palsies. MRI revealed the presence of granulation tissue at the osseocartilaginous junction. Histology from the right external auditory canal confirmed the presence of necrotizing acute inflammation. A multidisciplinary approach was used to treat the patient, which included intravenous antibiotics (ceftazidime), strict glycemic control, and right tarsorrhaphy. This resulted in significant improvement, with normalization of inflammatory markers and resolution of cranial nerve palsies, though hearing loss persisted. This case highlights the importance of early diagnosis, culture-guided antibiotic therapy, and a multidisciplinary treatment strategy in managing NOE, particularly in patients with diabetes mellitus. Extended monitoring is crucial to prevent recurrence.

## Introduction

Necrotizing otitis externa (NOE), historically known as malignant otitis externa (MOE), is an uncommon, severe, invasive infection affecting the external auditory canal (EAC), temporal bone, and skull base [1,2]. This infection originates from the EAC and spreads through the osseocartilaginous junction or fissures of Santorini into the skull base, causing osteitis, chondritis, periostitis, osteomyelitis, and craniopathies [1,2]. Zonnour et al. (2023) demonstrated that about one-third of patients with MOE develop cranial nerve palsies [3]. A thorough workup for NOE is essential to avoid serious potential complications such as meningitis, brain abscess, dural venous sinus thromboses, and death [1,2].

The diagnosis of NOE relies on a robust clinical suspicion combined with microbiology cultures and imaging studies such as CT and/or MRI [3]. Various laboratory tests, including C-reactive protein (CRP), erythrocyte sedimentation rate (ESR), and differential leukocyte count, are often conducted. However, there is limited evidence that these tests are sensitive for diagnosing NOE; instead, they are used for monitoring and assessing the efficacy of treatment [2,4,5]. Management of NOE usually involves individualized systemic antibiotic therapy, strict control of diabetes mellitus, periodic radiological assessment, and multidisciplinary cooperation among various specialties [4,5]. NOE is a serious condition in diabetic patients due to increased risk of cranial nerve involvement, higher mortality rates, prolonged hospitalization, and poor treatment responses associated with inadequate glycemic control.

## Case presentation

A 67-year-old man presented to the emergency department on account of loss of consciousness. He had a 15-year history of type 2 diabetes mellitus with suboptimal glycemic control and had been receiving biphasic isophane insulin (30/70) for the past two years, with 38 units in the morning and 30 units in the evening. His medical history also included essential hypertension and chronic pancreatitis, and he had a 45-pack-year smoking history. On arrival, his blood glucose level was 2.6 mmol/L. He regained consciousness upon correction of the hypoglycemia. An obvious right facial nerve palsy was noted, and the patient confirmed that this had been ongoing for three weeks. The patient was known to the Otorhinolaryngology Department (ENT), with bilateral otitis externa, otitis media, and mastoiditis. Histology of the polypoidal lesion noted in the right auditory canal showed necrotizing acute inflammation. The patient had undergone a recent admission with diabetic ketoacidosis.

The right ear pain was persistent, insidious in onset, and mild-to-moderate in intensity. There was minimal right ear discharge with reduced hearing in the affected ear. Before admission, this was treated with oral co-amoxiclav and neomycin/dexamethasone/acetic acid ear spray. On examination, erythematous skin changes were noted over the bilateral mastoid processes. Evaluation by the ENT team showed an intact tympanic membrane with possible middle ear effusions and granulation tissue in the EAC.  Right ear toileting and swab were performed, which yielded *Pseudomonas aeruginosa* isolates resistant to ciprofloxacin.

Given the right facial palsy, a full cranial nerve examination was performed. Significant changes were observed in some of the cranial nerves, as outlined in Table 1.

**Table 1:** Clinical findings from the cranial nerve examination.

Cranial nerves	Clinical findings
Opthalmic nerve	Blurry vision in the right eye due to exposure keratitis secondary to facial nerve palsy, with no evidence of ophthalmic nerve involvement
Oculomotor, trochlear, and abducens nerves	Double vision, especially on the left lateral gaze
Facial nerve	Right lower motor neuron type of facial palsy(House Brackmann grading, stage IV, moderately severe) and reduced sense of taste on the right half of the tongue, anterior two-thirds
Vestibulocochlear nerve	Gross bilateral hearing reduction was noted clinically; formal audiometry and stapedius reflex testing were not performed. Rinne’s and Weber’s tests were suggestive of conductive hearing loss
Glossopharyngeal and vagus nerve	Difficulty in swallowing and hoarseness of voice
Hypoglossal nerve	Tongue slightly deviated to the right side

**Figure 1:**
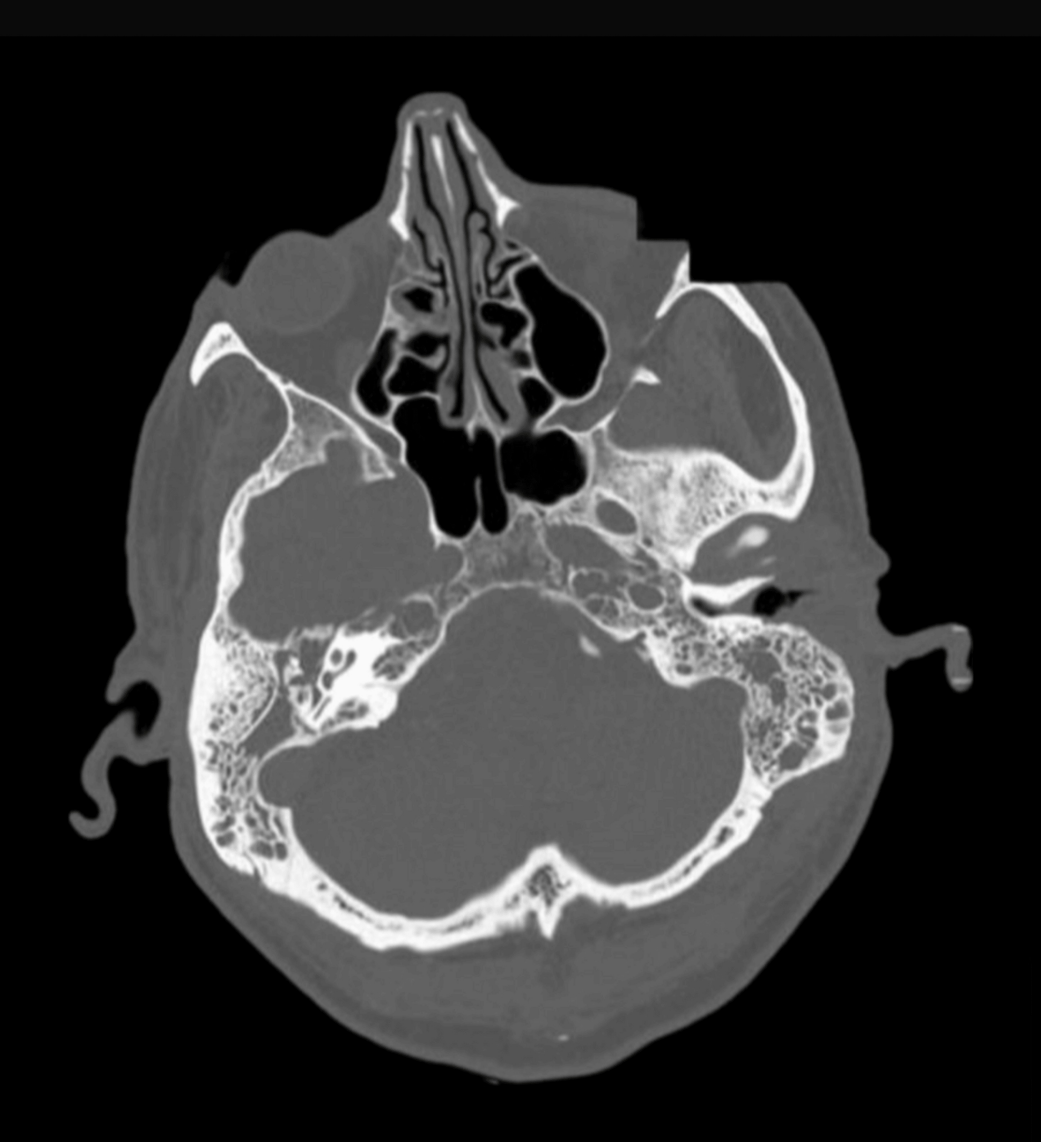
CT of the head showing tissue destruction of the right petrous apex and skull base.

**Figure 2:**
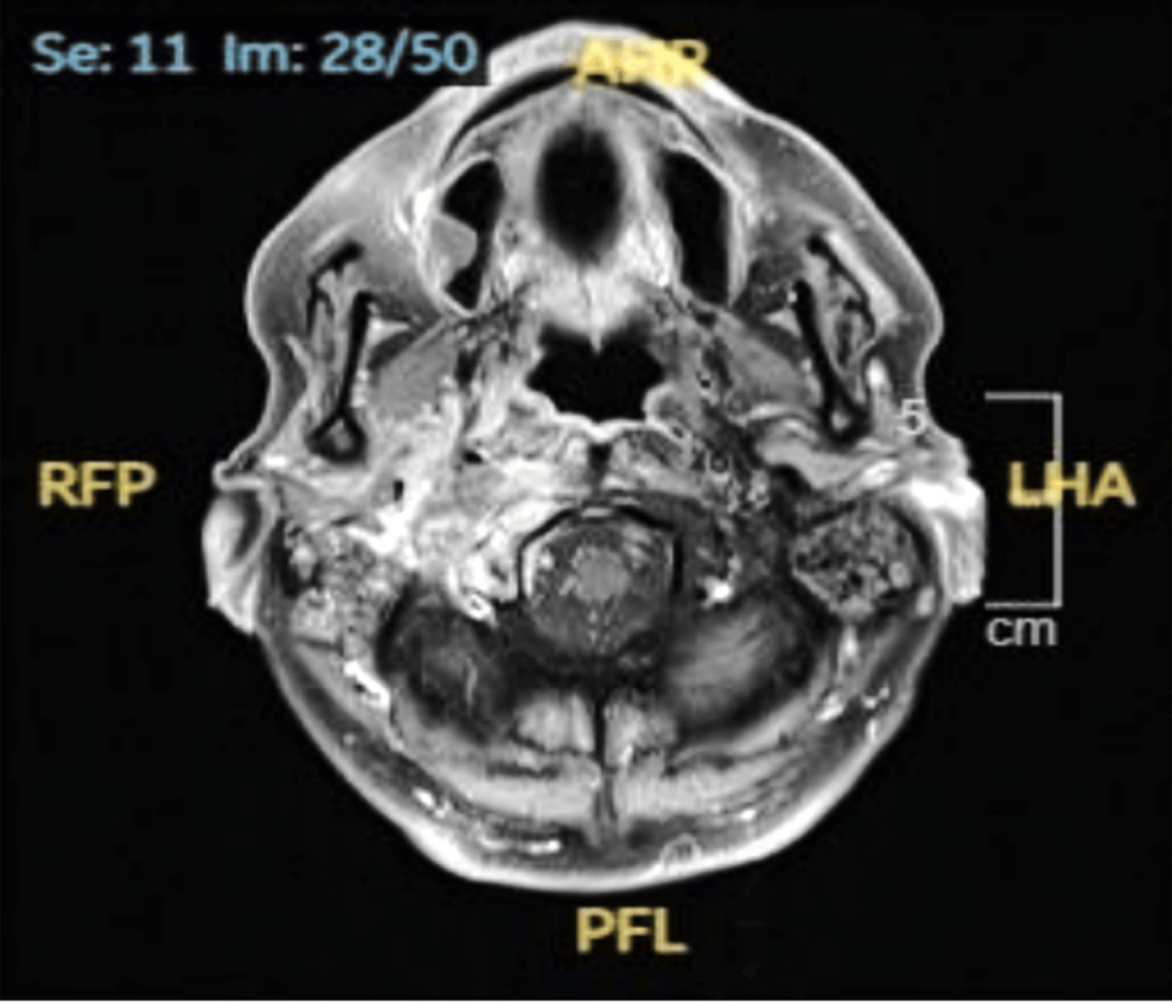
MRI of the head with contrast showing features of necrotizing external otitis on the right, with skull base osseous destruction/osteomyelitis, small mastoidal abscesses, and mastoiditis.

On laboratory investigation, his initial blood tests showed raised inflammatory markers, with CRP of 73 mg/L and white blood cell count (WBC) of 23.7 × 10^9^/L (neutrophilic predominance). In addition, his HbA1c value was 74 mmol/mol. A CT of the head upon admission showed rapidly progressive tissue destruction of the right petrous apex and skull base, with the overall appearance suggestive of an aggressive neoplastic lesion (Figure 1).  A subsequent MRI of the head (Figure 2) showed extensive right-sided NOE with skull base osteomyelitis consistent with complex NOE (UK consensus) and Carney Stage 3b (multiple cranial nerve involvement). An ultrasound Doppler of the carotids and jugular veins ruled out any thrombosis.

He was managed with frequent multidisciplinary inputs from various specialists, including microbiology, otorhinolaryngology, endocrinology, and ophthalmology. Though the initial empirical treatment with intravenous (IV) co-amoxiclav was later escalated to IV tazocin based on the microbiology input, antibiotics were further changed to IV ceftazidime for six weeks, in accordance with culture sensitivity and standard practice for skull base osteomyelitis duration.

Blood glucose levels were optimized by titrating his insulin regimen. The skull base multidisciplinary team ruled out a neoplastic process, confirmed the diagnosis as NOE with skull base osteomyelitis, and advised conservative management. However, the patient had to undergo a right tarsorrhaphy to treat the exposure keratitis.

Before discharge from the hospital, his ear pain and discharge had improved, with significant demonstrable improvement in the right-sided facial palsy. Inflammatory markers were back to normal, with CRP of 1.4 g/L and WBC of 8.7 × 10^9^/L. On follow-up 10 days after discharge, both the right and left ear tympanic membranes were intact, without any signs of infection or granulation on otoscopy, and resolved cranial nerve palsy, although there was persistent hearing loss.

## Discussion

NOE is a serious infection of the external ear canal and lateral skull base. In the past, the word *malignant* was used to describe the rapid spread and poor outcome, but the condition is unrelated to cancer; hence, the term *necrotizing* has been adopted in recent years, although neither term accurately describes the pathophysiology [2,4,6]. NOE is often under-recognized and misdiagnosed, particularly in diabetic patients. It is crucial to comprehend the disease etiology and diagnostic criteria due to its poor prognosis.

The most common causative organism for NOE is reported to be the gram-negative bacterium *Pseudomonas aeruginosa* [2,4]. Other bacterial causes include *Staphylococcus epidermidis* and *Klebsiella species*. In patients without diabetes mellitus, methicillin-resistant *Staphylococcus aureus* should be considered [4]. Fungal cases associated with *Aspergillus fumigatus* and *Candida* are also reported, and patients with human immunodeficiency virus (HIV) are more prone to fungal infections [4].

NOE is commonly reported in the elderly and immunocompromised people. There is a strong male predisposition, and most patients are found to have diabetes mellitus, especially with suboptimal glycemic control [2,6]. Although there is no difference in the prevalence between type 1 and type 2 diabetes mellitus, those with type 1 diabetes mellitus tend to have a more adverse outcome [2]. On the contrary, there are also reports of NOE in younger patients with immunocompromise, such as HIV, recent chemotherapy, and the use of immunosuppressive medication [2,7].

The 2023 UK consensus for NOE summarizes the difference between possible, definite, and complex NOE, as depicted in Table 2 [6]. Before that, the 1987 Cohen and Friedman Criteria (Table 3) [2] were widely used, alongside the Carney Clinicopathological Staging System for disease grading (Table 4) [4].

**Table 2:** UK Consensus for NOE. Table independently created by the authors using information from Hodgson et al. [6]. NOE = necrotizing otitis externa; EAC = external auditory canal

Definite NOE	Possible NOE	Complex NOE
Presence of all features	No radiological features of NOE, but the presence of all	Definite NOE and presence of
Otalgia and otorrhoea	Otalgia and otorrhoea	Facial and other lower cranial nerve involvement
Granulation or inflammation of the EAC	Granulation or inflammation of the EAC	Radiological evidence of cerebral venous thrombosis
Radiological features of NOE	Immunodeficiency, night pain, raised inflammatory markers, or failure of treatment	Extensive bone involvement

**Table 3:** 1987 Cohen and Friedman diagnostic criteria for NOE. For a diagnosis of NOE, all major criteria must be met, as the presence of only minor criteria is insufficient for the diagnosis. Table independently created by the authors using information from Al Aaraj and Kelley [2]. NOE = necrotizing otitis externa; EAC = external auditory canal

Major (obligatory) criteria	Minor (occasional) criteria
Disproportionate pain to EAC findings	History of diabetes mellitus
Edema	Involvement of the cranial nerve
Exudate	Positive radiological feature
Granulation of EAC	Debilitating condition
Micro-abscesses (if surgery performed)	Old age
Positive Tc-99m bone scan	
Failure to improve after one week of local treatment	

**Table 4:** Carney Clinicopathological Staging System for NOE. This table has been adapted from Tsilivigkos et al. [4], which is an open-access article distributed under the terms and conditions of a Creative Commons license. NOE = necrotizing otitis externa

Stages	Features
Stage 1	Clinical evidence of NOE with inflammation of soft tissues extending beyond the external auditory meatus
Stage 2	Clinical evidence of NOE with inflammation of soft tissues extending beyond the external auditory meatus and a positive Tc-99m bone scan
Stage 3	Clinical evidence of NOE with inflammation of soft tissues extending beyond the external auditory meatus, a positive Tc-99m bone scan, and cranial nerve involvement. Stage 3a: Single cranial nerve palsy. Stage 3b: Multiple cranial nerve palsies
Stage 4	Meningitis, dural venous sinus thrombosis, empyema, and brain abscess

The incidence of NOE is relatively rare, with reported cases of only up to 1.19 per 100,000 patients in national publications [2]. The study proposed that the huge increase in incidence could be multifactorial, caused by an increase in the prevalence of diabetes mellitus, an increasing geriatric population, increasing awareness/recognition, and accurate coding of the diagnosis. The new UK Consensus developed by experts in the field provides a clear stratification to better guide future diagnosis of NOE. Based on these criteria, it is possible that this is a complex case of NOE, meeting at least two out of three criteria.

A recent study from Cambridge University documented a 1,142% increase in the incidence of NOE over 16 years [8]. The progression of this case took about three weeks, from the onset of symptoms to the development of complications. This seems to be the typical course of NOE, as seen in other cases [1,9]. This case presented a multitude of similarities to several previous case reports, including being a male and having a history of diabetes mellitus with suboptimal control [1,4,5], as reflected in the patient’s HbA1c value. The prevailing explanation is that diabetes mellitus exacerbates NOE by causing small vessel vasculopathy and immune dysfunction [2]. It also changes the pH of the cerumen in the ear, which predisposes the patient to ear infection [1,5].

The cranial neuropathies in most studies include the facial and vestibulocochlear nerves [5], although this case had a more uncommon involvement of the glossopharyngeal/vagus and hypoglossal nerves.  Based on the Carney Clinicopathological Classification (Table 4), this qualifies for at least Grade 3b severity due to the involvement of multiple cranial nerves.  The presence of the lower cranial nerve palsies is thought to be a poor prognostic factor [5].

A multidisciplinary approach to management was adopted as the patient developed ophthalmic, ENT, and neurological complications. The pharmacological treatment was in line with the mainstay of treatment, which is IV antibiotics/antifungals, guided by microbial culture and sensitivity analysis [2]. A prolonged course of the IV anti-pseudomonal drug, ceftazidime, was administered in place of the widely used ciprofloxacin [4], due to antimicrobial resistance. Additional management consisted of good glycemic control and right tarsorrhaphy, with a serial assessment of response to treatment using inflammatory markers, otoscopy, and imaging (cranial MRI).

The patient’s response to treatment was as expected from previous studies. It is noteworthy that most case studies involving NOE were treated with ciprofloxacin. Although the second line of antibiotic therapy was used as per culture/sensitivity, the symptomatic improvement demonstrated its effectiveness. Further research into the use of ceftazidime in NOE and its comparison with ciprofloxacin might be beneficial. Evidence from the literature suggests that patients with NOE should be followed up for at least one year after therapy, due to the high risk of recurrence [10,11]. A minimum period of three months is required to be identified as cured [11].

Hyperbaric oxygen therapy (HBOT) may be a valuable adjunct in refractory or advanced NOE, particularly in cases with cranial nerve involvement. Byun et al. (2020) reviewed 16 studies involving 58 patients with NOE treated with adjunctive HBOT, most of whom had diabetes mellitus and *Pseudomonas aeruginosa* infection. Cranial nerve VII was involved in 55.2% of patients. The overall cure rate with HBOT was 91.4%, with all-cause mortality of 8.6%, and 72% of patients with facial nerve palsy recovered function [12]. However, its efficacy remains unproven due to the lack of randomized controlled trials.

## Conclusions

This case report underscores the critical need for early recognition and comprehensive management of NOE in patients with diabetes mellitus. The patient with type 2 diabetes mellitus with suboptimal glycemic control developed severe NOE complicated by multiple cranial nerve palsies. Timely diagnosis through imaging and microbiological culture, coupled with a multidisciplinary treatment approach involving targeted antibiotics, glycemic control, and surgical intervention, led to a positive outcome. The case highlights the importance of culture-guided antibiotic therapy, vigilant neurological assessment, and prolonged follow-up to prevent recurrence. This report contributes to understanding the implications of NOE and the necessity for coordinated care.
